# 4-(3,4-Diacetyl-5-methyl-1*H*-pyrazol-1-yl)benzene­sulfonamide

**DOI:** 10.1107/S1600536811005733

**Published:** 2011-02-23

**Authors:** Hatem A. Abdel-Aziz, Ahmed Bari, Seik Weng Ng

**Affiliations:** a Department of Pharmaceutical Chemistry, College of Pharmacy, King Saud University, Riyadh 11451, Saudi Arabia; b Department of Chemistry, University of Malaya, 50603 Kuala Lumpur, Malaysia

## Abstract

In the title mol­ecule, C_14_H_15_N_3_O_4_S, the pyrazole ring is aligned at a dihedral angle of 55.5 (1)° with respect to the benzene ring; the mean planes of the acetyl substituents are twisted by 13.4 (3) and 30.1 (3)° with respect to the pyrazole ring. Inter­molecular classical N—H⋯O and weak C—H⋯O hydrogen bonding links the mol­ecules, forming a three-dimensional network architecture in the crystal structure.

## Related literature

For background to the biological properties of aryl-substituted pyrazoles, see: Abdel-Aziz *et al.* (2010 ▶).
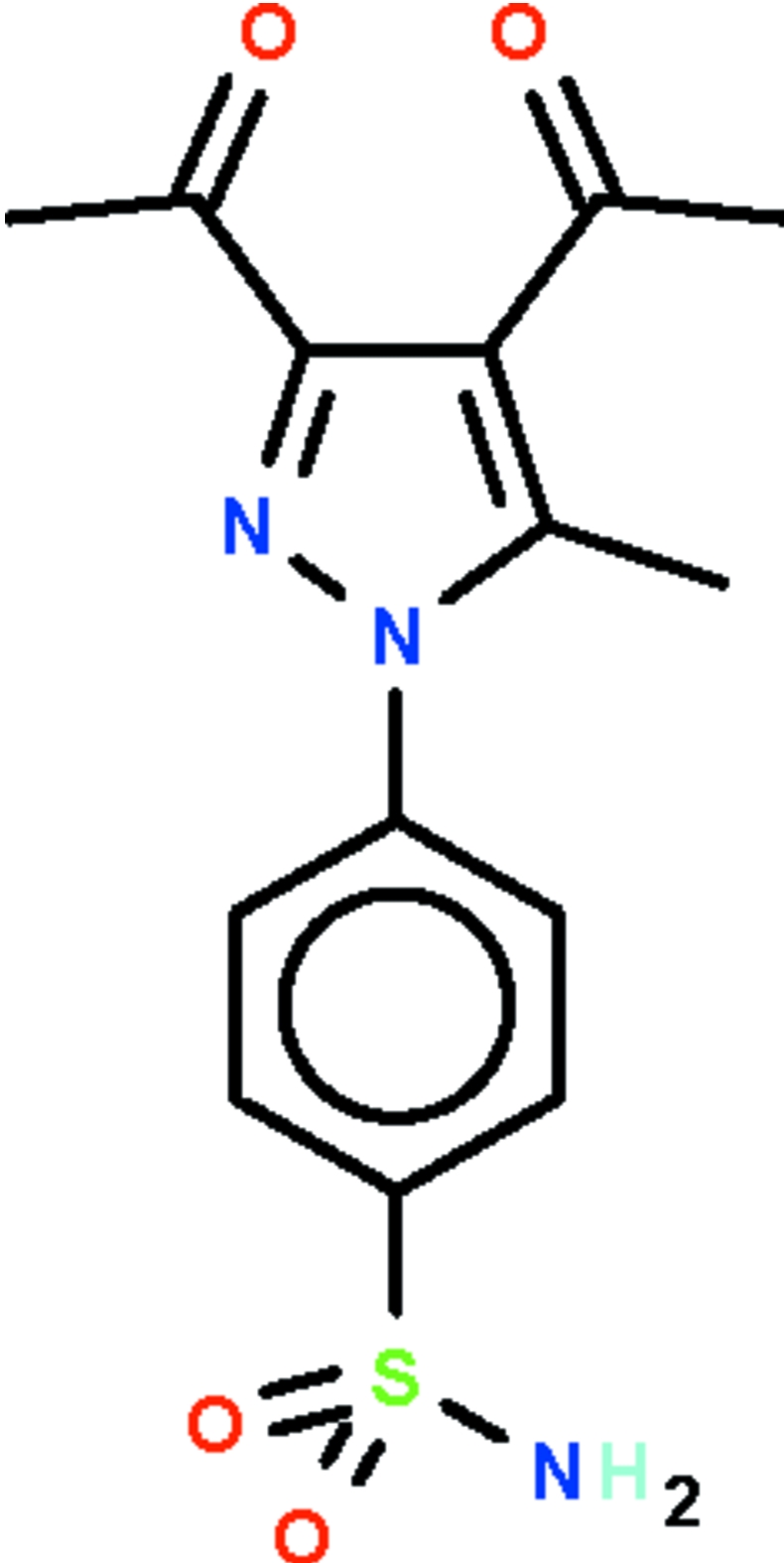


## Experimental

### 

#### Crystal data

C_14_H_15_N_3_O_4_S
*M* *_r_* = 321.35
Orthorhombic, 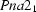

*a* = 8.3716 (3) Å
*b* = 21.7722 (8) Å
*c* = 7.8915 (3) Å
*V* = 1438.37 (9) Å^3^
*Z* = 4
Mo *K*α radiation
μ = 0.25 mm^−1^
*T* = 100 K
0.20 × 0.15 × 0.05 mm

#### Data collection

Agilent SuperNova Dual diffractometer with an Atlas detector
Absorption correction: multi-scan (*CrysAlis PRO*; Agilent, 2010 ▶) *T*_min_ = 0.952, *T*_max_ = 0.988
10477 measured reflections
3087 independent reflections
2634 reflections with *I* > 2σ(*I*)
*R*_int_ = 0.056

#### Refinement

*R*[*F* ^2^ > 2σ(*F* ^2^)] = 0.042
*wR*(*F* ^2^) = 0.095
*S* = 1.05
3087 reflections
210 parameters
3 restraints
H atoms treated by a mixture of independent and constrained refinement
Δρ_max_ = 0.31 e Å^−3^
Δρ_min_ = −0.39 e Å^−3^
Absolute structure: Flack (1983 ▶), 1337 Friedel pairs
Flack parameter: 0.08 (8)

### 

Data collection: *CrysAlis PRO* (Agilent, 2010 ▶); cell refinement: *CrysAlis PRO*; data reduction: *CrysAlis PRO*; program(s) used to solve structure: *SHELXS97* (Sheldrick, 2008 ▶); program(s) used to refine structure: *SHELXL97* (Sheldrick, 2008 ▶); molecular graphics: *X-SEED* (Barbour, 2001 ▶); software used to prepare material for publication: *publCIF* (Westrip, 2010 ▶).

## Supplementary Material

Crystal structure: contains datablocks global, I. DOI: 10.1107/S1600536811005733/xu5161sup1.cif

Structure factors: contains datablocks I. DOI: 10.1107/S1600536811005733/xu5161Isup2.hkl

Additional supplementary materials:  crystallographic information; 3D view; checkCIF report

## Figures and Tables

**Table 1:** Hydrogen-bond geometry (Å, °)

*D*—H⋯*A*	*D*—H	H⋯*A*	*D*⋯*A*	*D*—H⋯*A*
N3—H31⋯O2^i^	0.88 (1)	2.03 (1)	2.864 (3)	159 (3)
N3—H32⋯O4^ii^	0.88 (1)	2.06 (1)	2.933 (3)	170 (3)
C1—H1*C*⋯O3^i^	0.98	2.55	3.446 (3)	151
C10—H10⋯O1^iii^	0.95	2.51	3.314 (3)	142
C14—H14⋯O1^iv^	0.95	2.54	3.414 (3)	153

Symmetry codes: (i) 
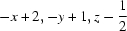
; (ii) 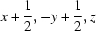; (iii) 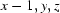; (iv) 
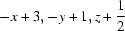
.

**Fig. 1.**
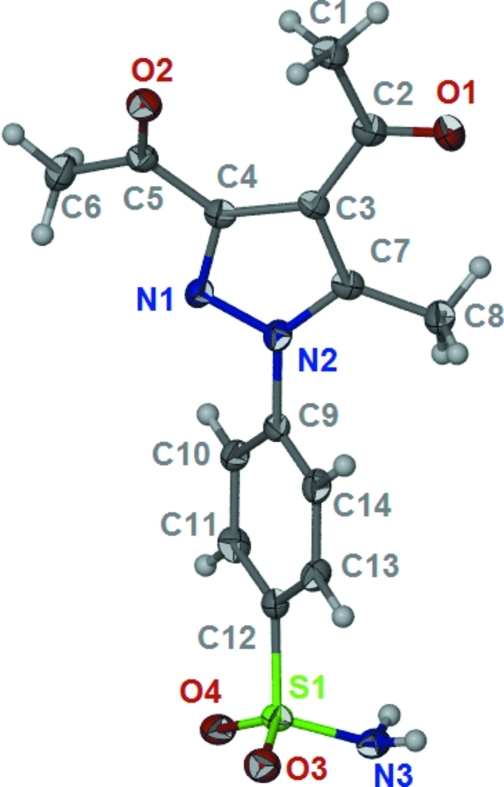
Thermal ellipsoid plot (Barbour, 2001) of C14H15N3O4S at the 70% probability level; hydrogen atoms are drawn as spheres of arbitrary radius.

## supplementary crystallographic information

### Comment

We have reported the antitumor activity of aryl-pyrazoles against CaCo-2 and HEP-2 cell lines (Abdel-Aziz *et al.*, 2010). These compounds were synthesized by a cycloaddition under microwave conditions. The present study involves the synthesis of an aryl-pyrazole having a sulfonamide –SO_2_NH_2_ substituent (Scheme I) that is expected to improve aqueous solubility. The C_14_H_15_N_3_O_4_ molecule has two acetyl substitutents on the pyrazolyl ring along with a benzenesulfanomide group. The sulfonamido unit interacts with an adjacent acetyl and sulfonamido O-atoms to generate a three-dimensional network (Table 1).

### Experimental

1-Phenyl-2-(phenylsulfonyl)ethanone (0.26 g, 10 mmol) was dissolved in a sodium ethoxide solution (prepared by dissolving 0.23 g sodium metal in 50 ml absolute ethanol). To the solution was added (*Z*)-2-oxo-*N*'-(4-sulfamoylphenyl)propanehydrazonoyl chloride (0.28 g, 10 mmol). The mixture was stirred for 12 h. The mixuture was then poured into cold water; the solid product was collected and recrystallized from an ethanol-water (4:1) mixture.

### Refinement

Carbon-bound H-atoms were placed in calculated positions (C–H 0.95–0.98 Å) and were included in the refinement in the riding model approximation, with *U*_iso_(H) set to 1.2–1.5 times *U*_eq_(C).

The amino H-atoms were located in a difference Fourier map, and were refined with a distance restraint of N–H 0.88±0.01 Å; their temperature factors were refined.

### Crystal data

**Table:**

C_14_H_15_N_3_O_4_S	*F*(000) = 672
*M**_r_* = 321.35	*D*_x_ = 1.484 Mg m^−^^3^
Orthorhombic, *P**n**a*2_1_	Mo *K*α radiation, λ = 0.71073 Å
Hall symbol: P 2c -2n	Cell parameters from 3291 reflections
*a* = 8.3716 (3) Å	θ = 2.4–29.2°
*b* = 21.7722 (8) Å	µ = 0.25 mm^−^^1^
*c* = 7.8915 (3) Å	*T* = 100 K
*V* = 1438.37 (9) Å^3^	Prism, colorless
*Z* = 4	0.20 × 0.15 × 0.05 mm

### Data collection

**Table:**

Agilent SuperNova Dual diffractometer with an Atlas detector	3087 independent reflections
Radiation source: SuperNova (Mo) X-ray Source	2634 reflections with *I* > 2σ(*I*)
Mirror	*R*_int_ = 0.056
Detector resolution: 10.4041 pixels mm^-1^	θ_max_ = 27.5°, θ_min_ = 2.6°
ω scans	*h* = −10→10
Absorption correction: multi-scan (*CrysAlis PRO*; Agilent, 2010)	*k* = −28→27
*T*_min_ = 0.952, *T*_max_ = 0.988	*l* = −10→9
10477 measured reflections	

### Refinement

**Table:**

Refinement on *F*^2^	Secondary atom site location: difference Fourier map
Least-squares matrix: full	Hydrogen site location: inferred from neighbouring sites
*R*[*F*^2^ > 2σ(*F*^2^)] = 0.042	H atoms treated by a mixture of independent and constrained refinement
*wR*(*F*^2^) = 0.095	*w* = 1/[σ^2^(*F*_o_^2^) + (0.0422*P*)^2^] where *P* = (*F*_o_^2^ + 2*F*_c_^2^)/3
*S* = 1.05	(Δ/σ)_max_ = 0.001
3087 reflections	Δρ_max_ = 0.31 e Å^−^^3^
210 parameters	Δρ_min_ = −0.39 e Å^−^^3^
3 restraints	Absolute structure: Flack (1983), 1337 Friedel pairs
Primary atom site location: structure-invariant direct methods	Flack parameter: 0.08 (8)

### Fractional atomic coordinates and isotropic or equivalent isotropic displacement parameters (Å^2^)

**Table:**

	*x*	*y*	*z*	*U*_iso_*/*U*_eq_	
S1	0.69022 (7)	0.29885 (3)	0.49994 (9)	0.01726 (16)	
O1	1.5767 (2)	0.55022 (9)	0.1918 (3)	0.0278 (5)	
O2	1.3205 (2)	0.68881 (8)	0.5594 (3)	0.0229 (5)	
O3	0.7216 (2)	0.27511 (9)	0.6664 (3)	0.0234 (5)	
O4	0.5323 (2)	0.31899 (7)	0.4576 (3)	0.0226 (5)	
N1	1.0863 (2)	0.56565 (9)	0.4491 (3)	0.0164 (5)	
N2	1.1261 (2)	0.50985 (9)	0.3847 (3)	0.0160 (5)	
N3	0.7369 (3)	0.24582 (10)	0.3684 (3)	0.0182 (5)	
H31	0.726 (3)	0.2569 (13)	0.2624 (17)	0.017 (8)*	
H32	0.8310 (18)	0.2289 (11)	0.385 (4)	0.021 (8)*	
C1	1.5821 (3)	0.64151 (12)	0.3508 (4)	0.0235 (7)	
H1A	1.6916	0.6438	0.3072	0.035*	
H1B	1.5845	0.6411	0.4750	0.035*	
H1C	1.5215	0.6773	0.3115	0.035*	
C2	1.5041 (3)	0.58395 (12)	0.2877 (4)	0.0196 (6)	
C3	1.3404 (3)	0.56637 (11)	0.3441 (3)	0.0149 (6)	
C4	1.2165 (3)	0.60007 (11)	0.4274 (3)	0.0157 (6)	
C5	1.2032 (3)	0.66253 (11)	0.5028 (4)	0.0179 (5)	
C6	1.0404 (3)	0.68993 (11)	0.5136 (5)	0.0252 (6)	
H6A	1.0466	0.7299	0.5704	0.038*	
H6B	0.9704	0.6625	0.5783	0.038*	
H6C	0.9972	0.6954	0.3992	0.038*	
C7	1.2751 (3)	0.50868 (12)	0.3166 (3)	0.0170 (6)	
C8	1.3336 (3)	0.45309 (12)	0.2257 (4)	0.0236 (7)	
H8A	1.2448	0.4339	0.1646	0.035*	
H8B	1.3777	0.4238	0.3077	0.035*	
H8C	1.4170	0.4650	0.1449	0.035*	
C9	1.0197 (3)	0.45957 (11)	0.4118 (4)	0.0160 (6)	
C10	0.8629 (3)	0.46375 (12)	0.3568 (3)	0.0176 (6)	
H10	0.8261	0.4996	0.3002	0.021*	
C11	0.7607 (3)	0.41477 (12)	0.3856 (4)	0.0196 (6)	
H11	0.6525	0.4168	0.3498	0.024*	
C12	0.8176 (3)	0.36269 (11)	0.4670 (3)	0.0172 (6)	
C13	0.9744 (3)	0.35924 (11)	0.5236 (4)	0.0178 (6)	
H13	1.0112	0.3236	0.5810	0.021*	
C14	1.0763 (3)	0.40801 (11)	0.4958 (4)	0.0190 (6)	
H14	1.1839	0.4063	0.5338	0.023*	

### Atomic displacement parameters (Å^2^)

**Table:**

	*U*^11^	*U*^22^	*U*^33^	*U*^12^	*U*^13^	*U*^23^
S1	0.0148 (3)	0.0163 (3)	0.0207 (4)	−0.0022 (2)	0.0012 (3)	0.0006 (3)
O1	0.0200 (10)	0.0241 (10)	0.0392 (13)	0.0012 (8)	0.0106 (10)	−0.0016 (10)
O2	0.0238 (11)	0.0223 (10)	0.0226 (12)	−0.0058 (8)	0.0029 (8)	−0.0047 (9)
O3	0.0263 (10)	0.0229 (10)	0.0211 (12)	−0.0025 (9)	0.0023 (9)	0.0001 (9)
O4	0.0157 (9)	0.0188 (9)	0.0334 (14)	−0.0020 (7)	0.0016 (8)	0.0017 (9)
N1	0.0164 (11)	0.0135 (10)	0.0192 (13)	0.0009 (8)	0.0006 (9)	−0.0009 (9)
N2	0.0147 (11)	0.0159 (10)	0.0175 (12)	0.0008 (9)	−0.0015 (9)	−0.0025 (10)
N3	0.0192 (12)	0.0160 (11)	0.0192 (14)	−0.0004 (9)	−0.0017 (10)	0.0022 (11)
C1	0.0171 (13)	0.0244 (14)	0.0289 (18)	−0.0047 (11)	0.0023 (12)	−0.0023 (14)
C2	0.0170 (13)	0.0203 (14)	0.0215 (16)	0.0011 (11)	−0.0017 (12)	0.0043 (14)
C3	0.0143 (12)	0.0174 (13)	0.0132 (15)	−0.0004 (10)	−0.0010 (10)	0.0031 (11)
C4	0.0165 (12)	0.0173 (12)	0.0134 (14)	−0.0015 (10)	−0.0011 (11)	0.0011 (12)
C5	0.0242 (13)	0.0162 (12)	0.0134 (13)	−0.0016 (10)	0.0032 (13)	0.0016 (14)
C6	0.0255 (14)	0.0191 (13)	0.0312 (18)	0.0020 (11)	0.0024 (15)	−0.0052 (14)
C7	0.0155 (13)	0.0180 (13)	0.0176 (15)	−0.0002 (10)	−0.0017 (11)	0.0026 (12)
C8	0.0190 (13)	0.0197 (14)	0.0321 (19)	−0.0007 (11)	0.0051 (12)	−0.0050 (13)
C9	0.0163 (12)	0.0147 (12)	0.0170 (15)	−0.0027 (10)	0.0032 (11)	−0.0048 (11)
C10	0.0173 (13)	0.0151 (12)	0.0204 (16)	0.0021 (10)	0.0002 (11)	−0.0003 (12)
C11	0.0118 (12)	0.0241 (13)	0.0229 (16)	0.0013 (11)	−0.0031 (11)	−0.0012 (14)
C12	0.0165 (12)	0.0158 (12)	0.0193 (17)	0.0001 (10)	0.0031 (11)	−0.0017 (12)
C13	0.0179 (12)	0.0150 (12)	0.0207 (16)	0.0032 (10)	−0.0021 (11)	0.0007 (12)
C14	0.0157 (12)	0.0189 (12)	0.0224 (15)	0.0016 (10)	−0.0001 (13)	−0.0040 (13)

### Geometric parameters (Å, °)

**Table:**

S1—O4	1.4325 (18)	C4—C5	1.489 (3)
S1—O3	1.436 (2)	C5—C6	1.490 (3)
S1—N3	1.601 (3)	C6—H6A	0.9800
S1—C12	1.771 (2)	C6—H6B	0.9800
O1—C2	1.218 (3)	C6—H6C	0.9800
O2—C5	1.221 (3)	C7—C8	1.490 (4)
N1—C4	1.334 (3)	C8—H8A	0.9800
N1—N2	1.358 (3)	C8—H8B	0.9800
N2—C7	1.358 (3)	C8—H8C	0.9800
N2—C9	1.427 (3)	C9—C10	1.385 (3)
N3—H31	0.875 (10)	C9—C14	1.387 (4)
N3—H32	0.879 (10)	C10—C11	1.386 (4)
C1—C2	1.498 (4)	C10—H10	0.9500
C1—H1A	0.9800	C11—C12	1.388 (4)
C1—H1B	0.9800	C11—H11	0.9500
C1—H1C	0.9800	C12—C13	1.388 (3)
C2—C3	1.491 (4)	C13—C14	1.380 (3)
C3—C7	1.387 (3)	C13—H13	0.9500
C3—C4	1.430 (4)	C14—H14	0.9500
			
O4—S1—O3	119.48 (11)	C5—C6—H6B	109.5
O4—S1—N3	107.16 (12)	H6A—C6—H6B	109.5
O3—S1—N3	106.79 (13)	C5—C6—H6C	109.5
O4—S1—C12	106.35 (11)	H6A—C6—H6C	109.5
O3—S1—C12	107.84 (12)	H6B—C6—H6C	109.5
N3—S1—C12	108.90 (12)	N2—C7—C3	106.5 (2)
C4—N1—N2	104.7 (2)	N2—C7—C8	120.5 (2)
N1—N2—C7	112.94 (19)	C3—C7—C8	132.9 (2)
N1—N2—C9	118.5 (2)	C7—C8—H8A	109.5
C7—N2—C9	128.2 (2)	C7—C8—H8B	109.5
S1—N3—H31	113 (2)	H8A—C8—H8B	109.5
S1—N3—H32	115.2 (19)	C7—C8—H8C	109.5
H31—N3—H32	110 (3)	H8A—C8—H8C	109.5
C2—C1—H1A	109.5	H8B—C8—H8C	109.5
C2—C1—H1B	109.5	C10—C9—C14	121.8 (2)
H1A—C1—H1B	109.5	C10—C9—N2	119.6 (2)
C2—C1—H1C	109.5	C14—C9—N2	118.6 (2)
H1A—C1—H1C	109.5	C9—C10—C11	118.9 (2)
H1B—C1—H1C	109.5	C9—C10—H10	120.6
O1—C2—C3	119.3 (2)	C11—C10—H10	120.6
O1—C2—C1	119.6 (2)	C10—C11—C12	119.5 (2)
C3—C2—C1	121.1 (2)	C10—C11—H11	120.2
C7—C3—C4	104.5 (2)	C12—C11—H11	120.2
C7—C3—C2	123.3 (2)	C11—C12—C13	121.2 (2)
C4—C3—C2	132.2 (2)	C11—C12—S1	120.17 (19)
N1—C4—C3	111.3 (2)	C13—C12—S1	118.66 (19)
N1—C4—C5	113.6 (2)	C14—C13—C12	119.5 (2)
C3—C4—C5	135.0 (2)	C14—C13—H13	120.3
O2—C5—C4	120.9 (2)	C12—C13—H13	120.3
O2—C5—C6	121.8 (2)	C13—C14—C9	119.2 (2)
C4—C5—C6	117.2 (2)	C13—C14—H14	120.4
C5—C6—H6A	109.5	C9—C14—H14	120.4
			
C4—N1—N2—C7	−2.5 (3)	C4—C3—C7—C8	174.8 (3)
C4—N1—N2—C9	170.7 (2)	C2—C3—C7—C8	−3.5 (5)
O1—C2—C3—C7	11.2 (4)	N1—N2—C9—C10	57.7 (3)
C1—C2—C3—C7	−167.1 (3)	C7—N2—C9—C10	−130.3 (3)
O1—C2—C3—C4	−166.6 (3)	N1—N2—C9—C14	−121.2 (3)
C1—C2—C3—C4	15.0 (5)	C7—N2—C9—C14	50.9 (4)
N2—N1—C4—C3	1.4 (3)	C14—C9—C10—C11	−0.5 (4)
N2—N1—C4—C5	−175.3 (2)	N2—C9—C10—C11	−179.4 (2)
C7—C3—C4—N1	0.1 (3)	C9—C10—C11—C12	−0.6 (4)
C2—C3—C4—N1	178.3 (3)	C10—C11—C12—C13	1.4 (4)
C7—C3—C4—C5	175.9 (3)	C10—C11—C12—S1	−178.3 (2)
C2—C3—C4—C5	−6.0 (5)	O4—S1—C12—C11	−10.3 (3)
N1—C4—C5—O2	148.1 (3)	O3—S1—C12—C11	−139.6 (2)
C3—C4—C5—O2	−27.6 (5)	N3—S1—C12—C11	104.9 (2)
N1—C4—C5—C6	−29.0 (4)	O4—S1—C12—C13	169.9 (2)
C3—C4—C5—C6	155.3 (3)	O3—S1—C12—C13	40.7 (3)
N1—N2—C7—C3	2.7 (3)	N3—S1—C12—C13	−74.9 (2)
C9—N2—C7—C3	−169.8 (2)	C11—C12—C13—C14	−1.1 (4)
N1—N2—C7—C8	−174.3 (2)	S1—C12—C13—C14	178.6 (2)
C9—N2—C7—C8	13.2 (4)	C12—C13—C14—C9	0.0 (4)
C4—C3—C7—N2	−1.6 (3)	C10—C9—C14—C13	0.8 (4)
C2—C3—C7—N2	−179.9 (2)	N2—C9—C14—C13	179.7 (2)

### Hydrogen-bond geometry (Å, °)

**Table:**

*D*—H···*A*	*D*—H	H···*A*	*D*···*A*	*D*—H···*A*
N3—H31···O2^i^	0.88 (1)	2.03 (1)	2.864 (3)	159 (3)
N3—H32···O4^ii^	0.88 (1)	2.06 (1)	2.933 (3)	170 (3)
C1—H1C···O3^i^	0.98	2.55	3.446 (3)	151
C10—H10···O1^iii^	0.95	2.51	3.314 (3)	142
C14—H14···O1^iv^	0.95	2.54	3.414 (3)	153

Symmetry codes: (i) −*x*+2, −*y*+1, *z*−1/2; (ii) *x*+1/2, −*y*+1/2, *z*; (iii) *x*−1, *y*, *z*; (iv) −*x*+3, −*y*+1, *z*+1/2.
